# MicroRNA-645 is an oncogenic regulator in colon cancer

**DOI:** 10.1038/oncsis.2017.37

**Published:** 2017-05-15

**Authors:** S T Guo, X Y Guo, J Wang, C Y Wang, R H Yang, F H Wang, X Y Li, H Hondermarck, R F Thorne, Y F Wang, L Jin, X D Zhang, C C Jiang

**Affiliations:** 1 Department of Molecular Biology, Shanxi Cancer Hospital and Institute, Shanxi, China; 2 School of Biomedical Sciences and Pharmacy, University of Newcastle, Callaghan, New South Wales, Australia; 3 School of Environmental and Life Sciences, University of Newcastle, Callaghan, New South Wales, Australia; 4 Department of Pathophysiology, School of Preclinical and Forensic Medicine, Sichuan University, Chengdu, China; 5 School of Medicine and Public Health, The University of Newcastle, Callaghan, New South Wales, Australia

## Abstract

Despite advances in early diagnosis and the development of molecularly targeted therapy, curative treatment of colon cancer once it has metastasized is yet to be accomplished. This is closely associated with deregulated CRC cell proliferation and resistance to apoptosis. Here we reveal that upregulation of microRNA-645 (miR-645) through DNA copy number gain is responsible for enhanced proliferation and resistance to apoptosis in colon cancer. MiR-645 was upregulated in most colon cancer tissues related to adjacent normal mucosa. This appeared to be associated with amplification of a section of chromosome 20q13.13, where miR-645 is located. Inhibition of miR-645 reduced proliferation and enhanced sensitivity to apoptosis triggered by the chemotherapeutic drugs 5-fluorouracil and cisplatin in CRC cells, and retarded colon cancer xenograft growth. Conversely, overexpression of miR-645 in normal colon epithelial cells enhanced proliferation and triggered anchorage-independent cell growth. Although SRY-related HMG-box 30 (SOX30) was identified as a miR-645 target, its expression was only partially affected by miR-645, suggesting that miR-645 is a fine-tuning mechanism of SOX30 expression. Moreover, overexpression of SOX30 only moderately inhibited promotion of CRC cell proliferation by miR-645, indicating that miR-645 may have more targets that contribute to its pro-proliferation effect in colon cancer. Together, this study reveals that miR-645 can regulate oncogenesis in colon cancer with SOX30 being one of its targets.

## Introduction

Colon cancer is among the most deadly cancers and has a high incidence rate.^1, 2^ Despite recent advances in early diagnosis and the development of molecularly targeted therapeutic approaches, the survival of metastatic colon cancer patients remains disappointing.^3^ The resistance of CRC cells to systemic therapies is frequently associated with the aberrant activation of survival signaling, including pathways mediated by oncogenic mutations of *KRAS* and *BRAF*.^4, 5, 6, 7^ In addition, dysregulated microRNAs (miRs) have been implicated in regulating colon cancer pathogenesis by acting as oncogenic or tumor suppressive regulators.^8, 9^

MiRs functions as ‘fine-tuning’ mechanisms. Regulation of protein coding gene expression by miRs is sequence specific and occurs mostly through binding of miRs to the 3′-UTR (untranslated region) regions of their target. This functions to either target the transcripts for degradation, or to block the transcript from being translated.^10, 11^ The functional significance and target selectivity is highly specific to each miR, and additionally, the expression status and function of miRs can be critically dependent on the tissue and cell type involved.^12^ Although how this is determined has not yet been fully explained, miRs are commonly situated to fragile clusters in the genome, where alterations to the genome are frequent.^13, 14, 15^ Indeed, we have previously found that miR-497 functions as a tumor suppressor in colon cancers and downregulation of this miR is due to gene copy number reduction.^16^

SRY-related HMG-box 30 (SOX30) is a transcription factor from the SOX family that are encoded by *SOX* genes^17^ and participates in regulation of embryonic development through differentiation of spermatocytes and spermatogenesis.^18, 19^ The gene encoding SOX30 is hypermethylated in a variety of tissues in adult mice.^20, 21, 22^ More recently, SOX30 has been found to be tumor suppressive in lung cancer through transcriptional activation of p53.^23^ In accordance, high levels of SOX30 is correlated with favorable prognosis of patients with lung adenocarcinomas.^24^ However, the role of SOX30 and its regulation in other types of human cancers remain unclear.

We report in this study the differentially expressed miRs in colon cancer tissues compared with paired adjacent noncancerous mucosa. We report here that miR-645 is markedly upregulated in colon cancer through amplification of its DNA copy number and functions as an oncogenic regulator to promote proliferation and resistance to cell death of CRC cells. Moreover, we show that although SOX30 is targeted by miR-645, its expression is only moderately affected by the levels of miR-645 expression, and that it is only partially responsible for the oncogenic effect of miR-645 on CRC cells.

## Results

### Upregulation of MiR-645 is frequently detected in CRC cells

**Figure 1:**
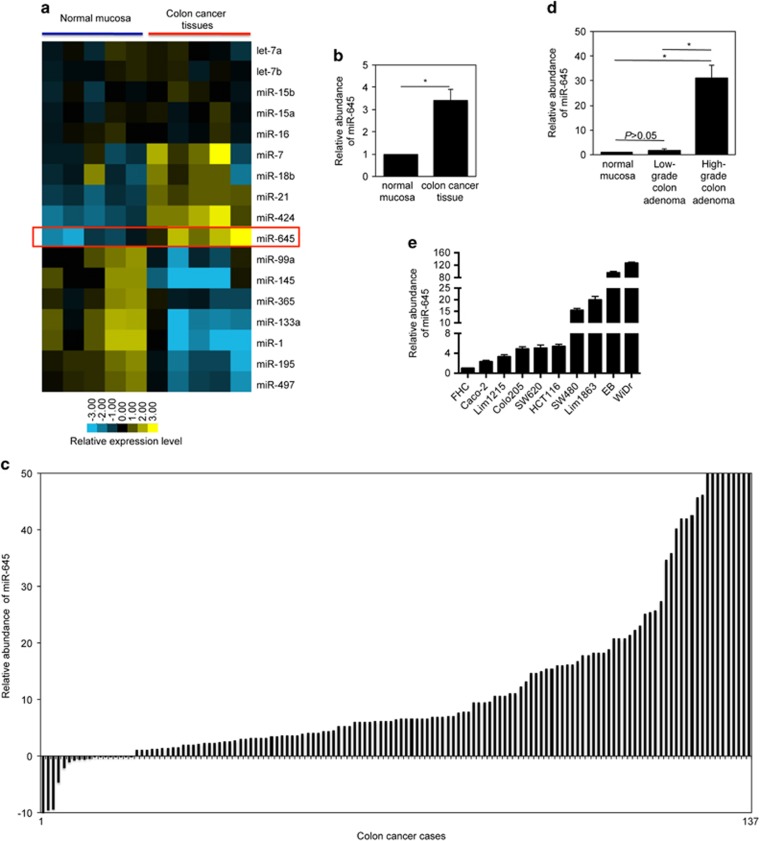
MiR-645 is upregulated in CRC cells. (**a**) MiR expression of 5 CRC tissues and corresponding control tissues by microarray analysis. Unsupervised hierarchical clustering was used for the array data analysis. (**b**) Quantitation of the microarray data showing that miR-645 is upregulated in CRC tissues. The data are presented as average fold changes of miR-645 level in CRC tissues normalized to their corresponding controls. *P<0.05, Student's *t*-test. (**c**) qPCR analysis showing that miR-645 is upregulated in the majority of colon cancers. The data shown are fold changes of miR-645 expression in CRC cases normalized to paired normal adjacent tissues. (**d**) qPCR analysis of miR-645 in high-grade colon adenomas, low-grade or normal colon epithelial tissues. The relative abundance of miR-645 in normal mucosa was arbitrarily designated as 1. mean±s.e., *n*=3. *P<0.05, Student's *t*-test. (**e**) qPCR analysis of miR-645 in cultured CRC cell lines and the FHC cells. The relative abundance of miR-645 in FHC cells was arbitrarily designated as 1. mean±s.e., *n*=3.

MiR profiles were compared between colon cancer tissues and adjacent normal mucosa from freshly removed surgical samples. The results showed that miR-645 was the only miR that was uniformly increased more than three-fold in each of the CRC tissues tested (Figures 1a, and Supplementary Tables 1,2).^16^ This increase in miR-645 expression was validated in an additional 137 pairs of CRC tissues by qPCR, indicating that miR-645 was upregulated to varying degrees in the vast majority colon cancer tissues (Figure 1c). Noticeably, no obvious difference was found in its expression between early and late stage CRC, nor among samples grouped according to anatomic origin, gender or age (Supplementary Table 3), suggesting that the increase of miR-645 is likely to be an early event that commonly occurs during CRC development. In support, the expression of miR-645 in low-grade colon adenomas was significantly lower compared with those of high-grade, whereas its expression levels were comparable between low-grade adenomas and normal mucosa (Figure 1d).

We then investigated the levels of miR-645 in cultured CRC cell lines by qPCR. In accordance with the results from CRC tissues, CRC cell lines displayed generally higher levels of miR-645 than the normal colon epithelial cell line FHC (Figure 1e).

### DNA copy number gain is responsible for upregulation of miR-645 in CRC cells

**Figure 2:**
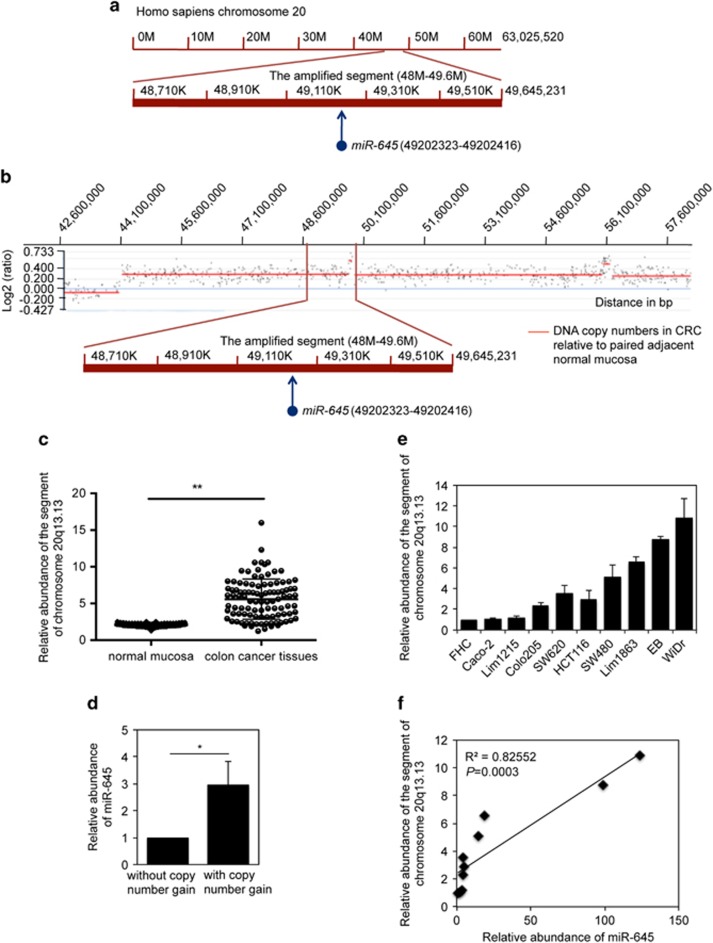
DNA copy number gain is responsible for the upregulation of miR-645 in CRC cells. (**a**) A schematic illustration showing that miR-645 is located to a segment (∼48–49.6 M) of chromosome 20q13.13. (**b**) Representative aCGH analysis indicating amplification of the 48–49.6 M segment of chromosome 20q13.13 in CRC tissues normalized to normal mucosa. (**c**) Genomic DNA from 100 pairs of CRC tissues was subjected to qPCR analysis, which confirms that the copy number of the 48–49.6 m segment of chromosome 20q13.13 is frequently amplified in CRC tissues. mean±s.e., *n*=3. **P<0.01, Student's *t*-test. (**d**) Total RNA from 100 pairs of CRC tissues was subjected to qPCR analysis, which shows that miR-645 is expressed at increased levels in CRC tissues with copy number gain in the 48–49.6  m segment of chromosome 20q13.13. The average level of miR-645 in CRC tissues without copy number gain of the 48–49.6 m segment was arbitrarily designated as 1. The average level of miR-645 in CRC tissues with copy number gain of the segment is expressed as the average fold of changes. mean±s.e., *n*=3. *P<0.05, Student's *t*-test. (**e**) Genomic DNA from cultured CRC cell lines and the FHC cells was subjected to qPCR analysis, which shows that the copy number of the 48–49.6 M segment of chromosome 20q13.13 is frequently amplified in CRC cell lines. The copy number of the fragment in FHC cells was arbitrarily designated as 1. mean±s.e., *n*=3. (**f**) Regression analysis indicating that miR-645 levels (fold changes in CRC cells relative to FHC cells) are correlated with copy number gain of the 48–49.6 M segment of chromosome 20q13.13 in CRC cells (R^2^=0.82552, *P*=0.0003).

Since regulation of miRNA expression is often due to alterations of the genome,^16^ we examined whether gene copy number changes are involved in the enhanced expression of miR-645 in CRC cells. MiR-645 is located to a segment (∼48–49.6 m) of chromosome 20q13.13 (Figure 2a), whereas copy number gain of chromosome 20q occurs in up to 70% of colon cancers.^25^ Indeed, copy number gain of chromosome 20q13.13 was found in 7 out of 10 randomly selected colon cancer tissue samples in comparison with paired normal tissue by aCGH (array comparative genomic hybridization) (Figure 2b). Genomic DNA from an additional 100 pairs of colon cancer tissues were analyzed by qPCR, which showed that 80 out of 100 CRC tissues had copy number amplification of the 48–49.6 m segment (Figure 2c). This suggests that upregulation of miR-645 in CRC tissues is due to its copy number gain. In support, colon cancer tissues with gain of the 48–49.6 m segment of chromosome 20q13.13 showed significantly higher levels of miR-645 compared with those without copy number changes in the segment (Figure 2d). Consistently, a correlation between DNA copy number gain of the 48–49.6 m segment and expression levels of miR-645 was also found in the majority of CRC cell lines (Figures 2e and f).

### MiR-645 promotes proliferation of CRC cells

**Figure 3:**
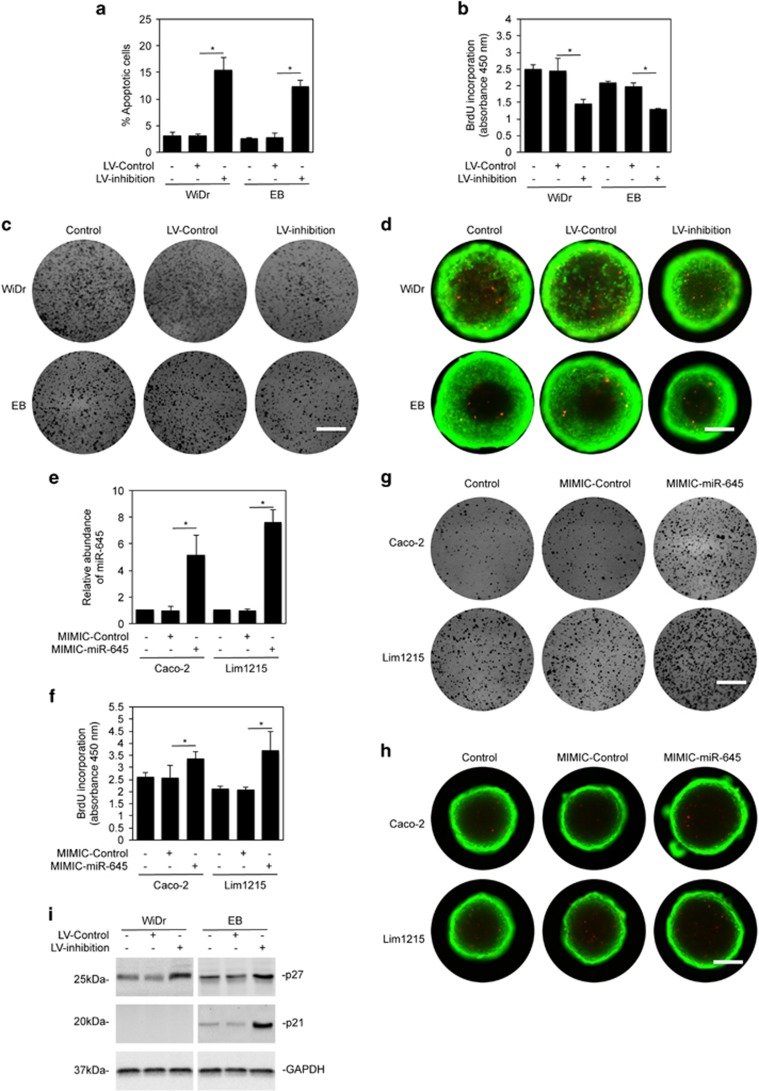
MiR-645 promotes proliferation in CRC cells. (**a**) WiDr and EB cells transduced with the miR-645 inhibitors (LV-inhibition) or the control (LV-Control) for 48 hours were subjected to quantitation of apoptosis. mean±s.e., *n*=3. *P<0.05, Student's *t*-test. (**b**) WiDr and EB cells transduced with the miR-645 inhibitors or the control were subjected to the BrdU incorporation assay. mean±s.e., *n*=3. *P<0.05, Student's *t*-test. (**c**) Viable cells of WiDr and EB cells transduced with miR-645 inhibitors or the control for 24 h were reseeded as single cell suspension onto 6-well plates. Cells were allowed to grow for 12 days followed with fixation with methanol and staining with crystal violet, *n*=3. Scale bar, 1cm. (**d**) Five hundred viable cells of WiDr and EB cells transduced with miR-645 inhibitors or the control for 24 h were seeded into a 96-well Perfecta3D Hanging drop plate for 12 days. Cells were then stained with calcein AM and ethidium homodimer-1 for 24 h, *n*=3. Green, viable cells; Red, dead cells. Scale bar, 25 μm. (**e**) Total RNA from Caco-2 and Lim1215 CRC cells transduced with the miR-645 mimics or the control was subjected to qPCR analysis. The abundance of miR-645 in cells without transduction was arbitrarily designated as 1. mean±s.e., *n*=3. *P<0.05, Student's *t*-test. (**f**) Caco-2 and Lim1215 cells transduced with the miR-645 mimics or the control for 48 h were subjected to the BrdU incorporation assay. mean±s.e., *n*=3. *P<0.05, Student's *t*-test. (**g**) Viable cells of Caco-2 and Lim1215 cells transduced with the miR-645 mimics or the control for 24 h were reseeded as single cell suspension onto 6-well plates. Cells were allowed to grow for 12 days followed with fixation with methanol and staining with crystal violet, *n*=3. Scale bar, 1 cm. (**h**) Five hundred viable cells of Caco-2 and Lim1215 cells transduced with the miR-645 mimics or the control for 24 h were seeded into a 96-well Perfecta3D Hanging drop plate for 12 days. Cells were then stained with calcein AM and ethidium homodimer-1 for 24 h, *n*=3. Green, viable cells; Red, dead cells. Scale bar, 25 μm. (**i**) Lysates from WiDr and EB cells transduced with the miR-645 inhibitors or the control were subjected to Western blot analysis, *n*=3.

To demonstrate the functional significance of upregulation of miR-645 in CRC cells, we stably transduced the miR-645 inhibitor or the scrambled control into cells of two widely used CRC cell lines, WiDr and EB, through lentiviral transduction.^16, 26^ While inhibition of miR-645 induced cell death in a relatively small proportion of CRC cells (around 10–15% apoptotic cells; Figure 3a), it resulted in inhibition of cell proliferation as demonstrated by the BrdU incorporation assay. This was further confirmed by the clonogenic assay (Figures 3b and c). Moreover, the growth of WiDr and EB cells in 3-dimensional (3D) cultures was also reduced when miR-645 was inhibited (Figure 3d). This indicates that miR-645 is involved in promoting CRC cell proliferation. In support, introduction of miR-645 mimics promoted BrdU incorporation, colony formation and cell growth in 3D cultures in Caco-2 and Lim1215 cells expressing relatively low endogenous miR-645 (Figures 1e and 3e–h). Inhibition of CRC cell proliferation by miR-645 inhibitor was associated with induction of p27 and p21, which are two well-established proteins that inhibit the cell cycle (Figure 3i), and cell cycle arrest in G1/G0 phase (Supplementary Figure 1).

### MiR-645 prevents CRC cells from undergoing apoptosis triggered by chemotherapeutic drugs

**Figure 4:**
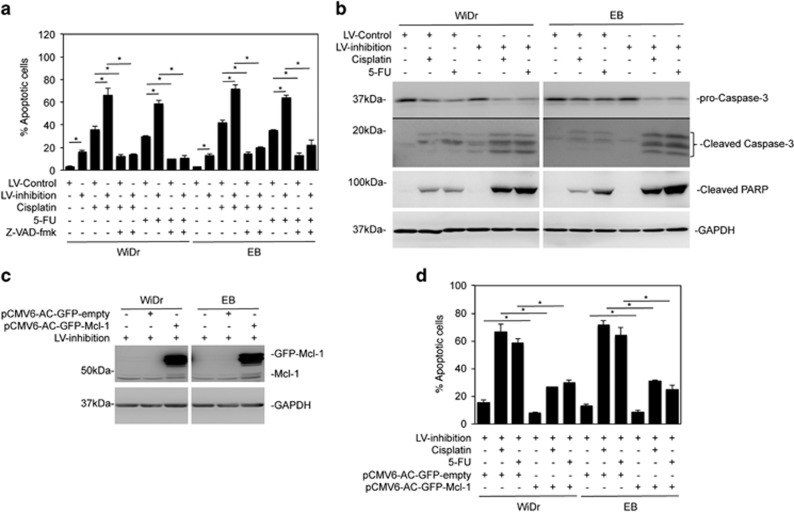
MiR-645 prevents CRC cells undergoing apoptosis. (**a**) WiDr and EB cells transduced with the miR-645 inhibitors or the control were pretreated with z-VAD-fmk (30 μM) for 1 h followed with the treatment of CDDP (5 μg/ml) or 5-FU (5 mg/ml) for an additional 48 h. Apoptosis was quantitated. mean±s.e., *n*=3. *P<0.05, Student's *t*-test. (**b**) Whole-cell lysates of WiDr and EB cells transduced with the miR-645 inhibitors or the control followed with the treatment with CDDP (5 μg/ml) or 5-FU (5 mg/ml) for 24 h were subjected to Western blot analysis, *n*=3. (**c**) WiDr and EB cells with miR-645 inhibition were transfected with the vector alone (pCMV6-AC-GFP-empty) or Mcl-1-GFP cDNA (pCMV6-AC-GFP-Mcl-1). Whole-cell lysates from were subjected to western blot analysis, *n*=3. (**d**) WiDr and EB cells with miR-645 inhibition were transfected with the vector alone or Mcl-1-GFP cDNA. Twenty-four hours later, cells were then treated with CDDP (5 μg/ml) or 5-FU (5 mg/ml) for a further 48 h. Apoptosis was quantitated. mean±s.e., *n*=3. *P<0.05, Student's *t*-test.

While inhibition of miR-645 did not significantly induce cell death (Figure 3a), it remains likely that miR-645 affects CRC cell sensitivity to killing triggered by other stimuli. To test this, WiDr and EB cells carrying the miR-645 inhibitor or the scrambled control were treated with the thymidylate synthase inhibitor 5-fluorouracil (5-FU) or the DNA-damaging drug cisplatin. Inhibition of miR-645 sensitized the cells to killing induced by both agents (Figure 4a). This was due to enhanced apoptosis, as z-VAD-fmk (a general caspase inhibitor) permitted sustained cell vitality in the presence of the cytotoxic drugs cisplatin and 5-FU in WiDr and EB cells with or without miR-645 inhibited (Figure 4a).^27, 28^ In support, introduction of anti-miR-645 enhanced the cleavage of caspase-3 and its substrate PARP due to cisplatin and 5-FU treatment (Figure 4b). Since cisplatin and 5-FU induce mitochondrion-mediated apoptosis in CRC cells,^27, 28^ which is inhibited by anti-apoptotic members of the Bcl-2 family such as Mcl-1,^29^ we tested the involvement of Mcl-1. Indeed, overexpression of Mcl-1 rescued the cells even when miR-645 was inhibited (Figures 4c and d). Collectively, these results indicate that miR-645 is involved in antagonizing caspase-dependent, mitochondrion-mediated apoptotic signalling in CRC cells.

### MiR-645 modulates colon cancer growth

**Figure 5:**
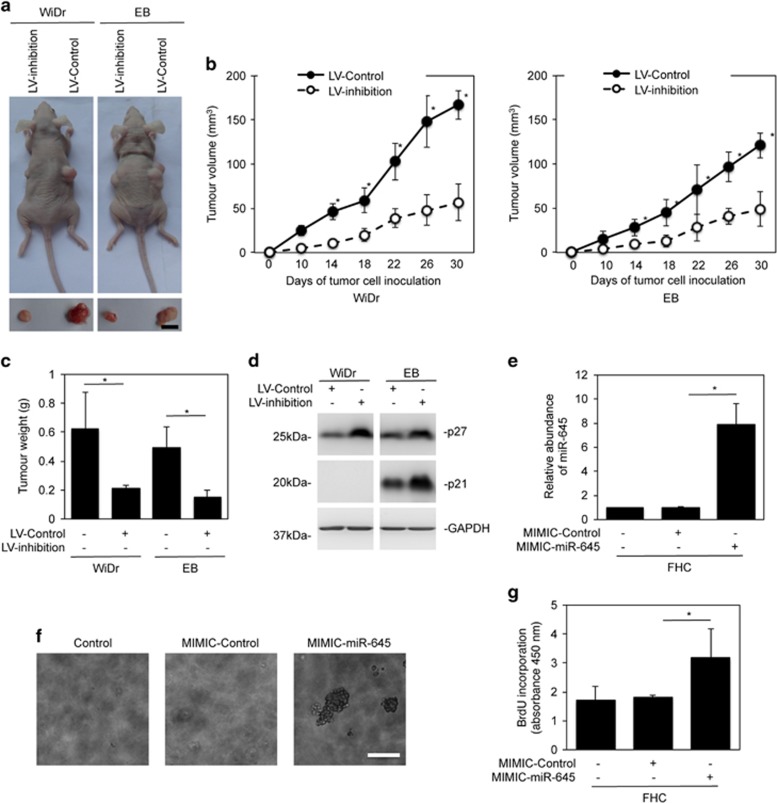
MiR-645 modulates colon cancer growth. (**a**) Representative photographs of xenografts of WiDr and EB cells with or without miR-645 knockdown. Scale bar, 5 mm. (**b**) Comparison of xenografts growth rates of WiDr (Left panel) and EB (Right panel) cells with or without miR-645 knockdown (*n*=8, mean±s.e., *P<0.05, Student's *t*-test). (**c**) Comparison of weights of harvested tumors of WiDr and EB cells with or without miR-645 knockdown. (*n*=8, mean±s.e., *P<0.05, Student's *t*-test). (**d**) Crude whole-cell lysates from xenografts of WiDr and EB cells with or without miR-645 knockdown were subjected to western blot analysis, *n*=3. (**e**) FHC cells were transduced with the miR-645 mimics or the control for 48 h. Total RNA was subjected to qPCR analysis of miR-645 expression. The abundance of miR-645 in cells without transduction was arbitrarily designated as 1. mean±s.e., *n*=3. *P<0.05, Student's *t*-test. (**f**) Overexpression of miR-645 caused anchorage-independent growth in FHC cells. Scale bar, 0.5 mm, *n*=3. (**g**) FHC cells were transduced with the miR-645 mimics or the control for 48 h. Cells were subjected to the BrdU incorporation assay. mean±s.e., *n*=3. *P<0.05, Student's *t*-test.

To reveal the effect of miR-645 on colon cancer growth *in vivo*, we xenografted WiDr and EB cells with or without stable lentiviral knockdown of miR-645 into nu/nu mice. Inhibited expression of miR-645 resulted in reduced growth of both WiDr and EB tumors (Figures 5a–c) along with increased expression of p27 and p21 (Figure 5d). Therefore, miR-645 can promote colon cancer growth *in vivo*, which is related to the enhanced cell cycle progression of CRC cells.

We also investigated whether miR-645 upregulation plays a role in transformation of normal colon epithelial cells by introducing miR-645 mimics into FHC cells (Figure 5e). Indeed, introduction of miR-645 mimics permitted anchorage-independent growth and resulted in an increased proliferative rate (Figures 5f and g). These observations are in accordance with the finding that miR-645 is upregulated during the early stages of CRC development (Supplementary Table 3 and Figure 1d).

### SOX30 is targeted by miR-645 in CRC cells

**Figure 6:**
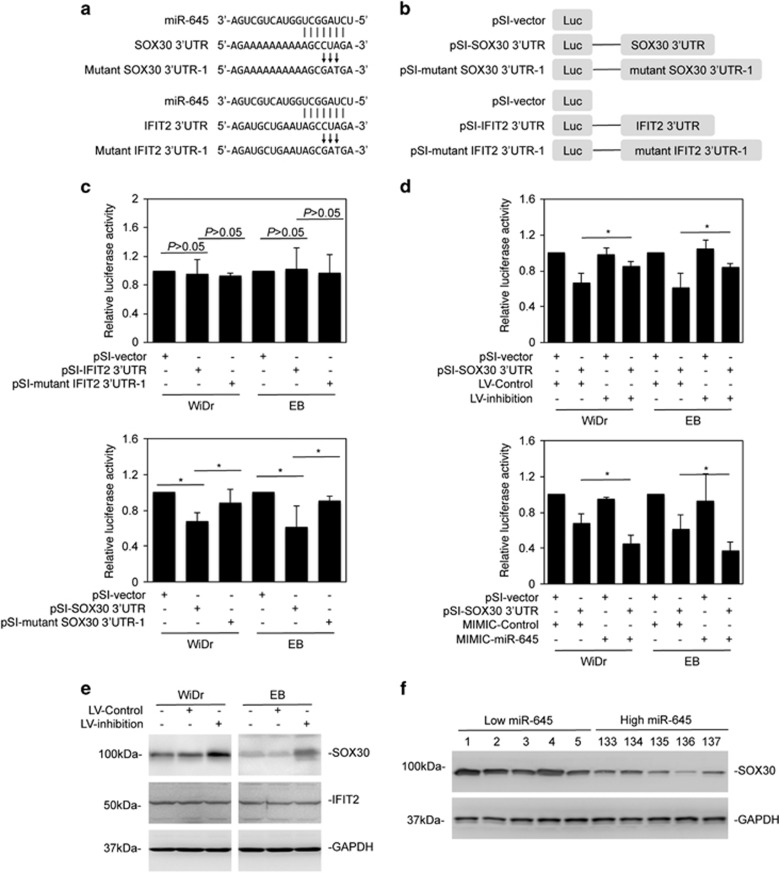
MiR-645 targets SOX30. (**a**) A schematic illustration of base-matching between miR-645 and the SOX30 3′-UTR (Upper panel) and the IFIT2 3′-UTR (Lower panel). Substitution of three consecutive bases (CUA to GAT) at the SOX30 or IFIT2 3′-UTR for mutant reporter constructs is also shown. (**b**) A schematic illustration of the luciferase reporter constructs. (**c**) The luciferase reporter activity of WiDr and EB cells transfected with the indicated reporter constructs was measured as mean±s.e., *n*=3. *P<0.05, Student's *t*-test. (**d**) Upper panel: WiDr and EB cells transduced with the miR-645 inhibitors or the control were introduced with the reporter constructs for 24 h. The luciferase reporter activity was measured. Lower panel: WiDr and EB cells transduced with the miR-645 inhibitors or the control were introduced with the reporter constructs for 24 h. The luciferase reporter activity was measured as mean±s.e., *n*=3. *P<0.05, Student's *t*-test. (**e**) Whole-cell lysates from WiDr and EB cells transduced with the miR-645 inhibitors or the control were subjected to Western blot analysis, *n*=3. (**f**) Western blot analysis of SOX30 in colon cancer samples #1–5 that expressed relatively low levels of miR-645 and #133–137 that expressed relatively high levels of miR-645 as shown in Figure 1c, *n*=3.

Having demonstrated that miR-645 has an oncogenic effect in CRC cells, potential targets of miR-645 were identified through searching the TargetScan (http://www.targetscan.org) and MicroRNA (http://www.microrna.org) databases. Among the targets found were the tumor suppressors SOX30 and Interferon-induced protein with tetratricopeptide repeats 2 (IFIT2) (Figure 6a).^23, 30^ MiR-645 has been reported to target IFIT2 in human adenocarcinoma of the gastric esophageal junction.^30^ To test whether miR-645 can affect the expression of IFIT2 and SOX30 in CRC cells, we cloned a 1901 bp fragment of the IFIT2 3′-UTR or a 661 bp fragment of the SOX30 3′-UTR, which contains the predicted binding region of miR-645 into a luciferase reporter construct (Figure 6b).^23, 30^ Unexpectedly, introduction of the construct into WiDr and EB cells showed that the presence of the 3′-UTR of IFIT2 did not significantly change the reporter activity compared with the basal level of the control vector (Figure 6c). On the other hand, the activity was inhibited by 30% with the presence of the SOX30 3′-UTR (Figure 6c). Mutation of the miR-645-binding region reversed the suppression of the reporter activity (Figure 6c and Supplementary Figure 2), suggesting that the endogenous expression of miR-645 attenuated the SOX30 3′-UTR activity. In support, the reporter activity was increased by co-introduction of miR-645 inhibitors, whereas it was further inhibited by the addition of miR-645 mimics (Figure 6d).

Consistently, inhibition of miR-645 through introduction of miR-645 inhibitors resulted in, albeit moderately, upregulation of the endogenous SOX30 protein levels, whereas the endogenous levels of the IFIT2 protein remained unaltered (Figure 6e). The correlation between the levels of miR-645 and SOX30 was also substantiated through examining sampled colon cancer tissues with relatively low (No. 1–5) and high (No. 133–137) levels of miR-645 (Figure 6f). MiR-645 expression was inversely correlated with the levels of SOX30 protein expression (Figure 6f). However, no association was found between the levels of SOX30 mRNA and the levels of miR-645 (Supplementary Figure 3). Together, these results indicate that miR-645 targets SOX30 in CRC cells, although the inhibitory effect of miR-645 on SOX30 level is only moderate.

### Suppression of SOX30 contributes to miR-645-mediated enhancement of cell proliferation and survival in CRC cells

**Figure 7:**
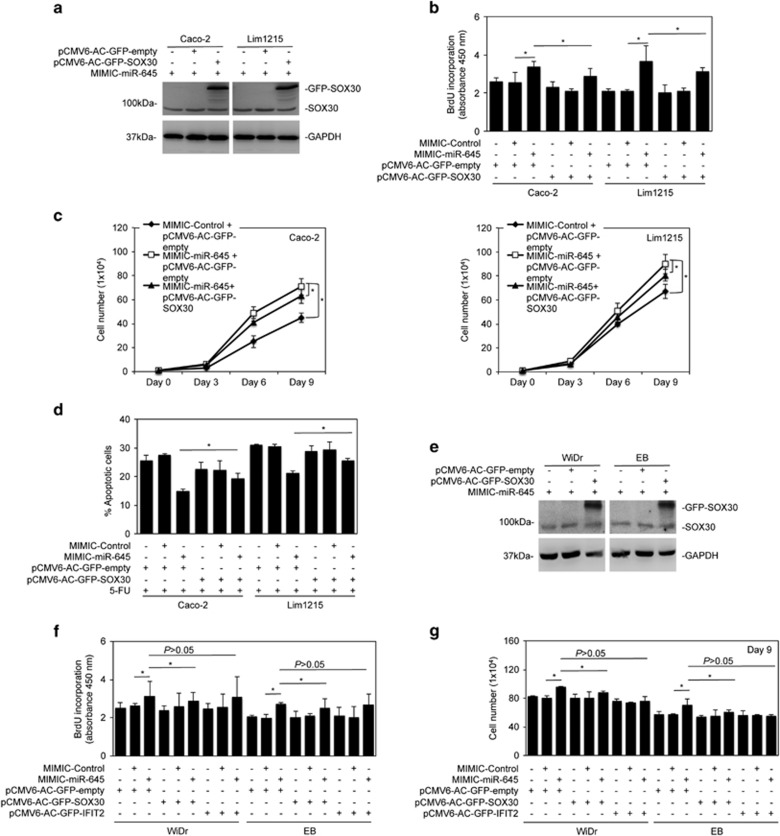
Suppression of SOX30 contributes to miR-645-mediated enhancement in proliferation and survival of CRC cells. (**a**) Caco-2 and Lim1215 cells transduced with miR-645 mimics were transfected with vector alone (pCMV6-AC-GFP-empty) or SOX30-GFP cDNA (pCMV6-AC-GFP-SOX30). Whole-cell lysates were subjected to western blot analysis, *n*=3. (**b**) Caco-2 and Lim1215 cells transduced with miR-645 mimics were transfected with vector alone or SOX30-GFP cDNA. Cells were subjected to the BrdU incorporation assay. mean±s.e., *n*=3. *P<0.05, Student's *t*-test. (**c**) Caco-2 (left) and Lim1215 (right) cells with stable overexpression of miR-645 were transfected with SOX30-GFP cDNA. Viable cells were counted at day 3, 6 and 9 after transfection. mean±s.e., *n*=3. *P<0.05, Student's *t*-test. (**d**) Caco-2 and Lim1215 cells transduced with miR-645 mimics were transfected with vector alone or SOX30-GFP cDNA followed the treatment with 5-FU (5 μg/ml) for 48 h. Apoptosis was quantitated. mean±s.e., *n*=3. *P<0.05, Student's *t*-test. (**e**) WiDr and EB cells transduced with miR-645 mimics were transfected with vector alone or SOX30-GFP cDNA. Whole-cell lysates were subjected to Western blot analysis, *n*=3. (**f**) WiDr and EB cells transduced with miR-645 mimics were transfected with vector alone, SOX30-GFP cDNA or IFIT2-GFP cDNA (pCMV6-AC-GFP-IFIT2). Cells were subjected to the BrdU incorporation assay. mean±s.e., *n*=3. *P<0.05, Student's *t*-test. (**g**) WiDr and EB cells transduced with miR-645 mimics were transfected with vector alone, SOX30-GFP cDNA or IFIT2-GFP cDNA (pCMV6-AC-GFP-IFIT2). Viable cells were counted at day 9 after transfection. mean±s.e., *n*=3. *P<0.05, Student's *t*-test.

We then investigated the functional significance of SOX30 in miR-645-mediated promotion of proliferation and survival of CRC cells by introducing a SOX30-expressing construct, which lacked the 3′-UTR and therefore could not be regulated by miRs targeting the 3′-UTR, along with the miR-645 mimics. As shown in Figures 7a–g, SOX30 overexpression moderately inhibited the promotion of cell proliferation and the increased level of apoptosis induced by 5-FU that is caused by miR-645 mimics. Nevertheless, the inhibitory effects were statistically significant (Figures 7b–d). This suggests that although SOX30 inhibition contributes to miR-645-mediated CRC cell proliferation and survival, other unidentified targets of miR-645 may be more critical in executing the biological function of miR-645 in colon cancer.

## Discussion

We have provided evidence in this report that miR-645 has an oncogenic role to promote proliferation and resistance to apoptosis in CRC cells. While miR-645 is commonly upregulated in CRC tissues, inhibition of miR-645 caused reduced CRC cell proliferation and increased sensitivity to induction of cell death. Our results also demonstrate the association between the upregulation of miR-645 and the amplification of the 48–49.6 M segment of chromosome 20q13.13, at which miR-645 is located. Noticeably, although miR-645 targets SOX30, its oncogenic effect is only partially accounted for its impact on SOX30, indicating that other unidentified targets are involved in executing its role in CRC cells.

Overall survival of patients with advanced ovarian cancer or head or neck squamous cell carcinoma (HNSCC) is negatively associated with the miR-645 expression level,^31, 32^ suggesting that it may have a role in the pathogenesis of these malignancies. However, it was not demonstrated until recently that miR-645 has an effect on cancer cell proliferation, survival and resistance to treatment.^23, 30^ For example, upregulation of miR-645 in gastric cancer cells promoted cell proliferation and protected cells from apoptosis.^30^ In addition, miR-645 increased invasion and metastasis of HNSCC. Similarly, our results demonstrated that in CRC cells miR-645 had an oncogenic role, as its inhibition reduced cell proliferation and rendered cells more sensitive to apoptosis induced by 5-FU and cisplatin. Although we clearly demonstrated that upregulation of miR-645 was found in the majority of colon cancers compared to paired noncancerous mucosa, we were unable to draw a conclusion as to whether increased miR-645 expression is important in predicting progression of disease and prognosis of patients, as the majority of patients involved were still alive. Of note, the differential expression of several miRsl, including miR-21, let-7, and miR-18b in CRC tissues has been demonstrated to have potential as prognostic biomarkers.^33, 34, 35^ Our finding showing that upregulation of miR-645 occurs early in the development of colon cancer suggests that testing its expression may be useful as a diagnostic biomarker for colon cancer.

Although miR-645 regulates gastric cancer cell proliferation and survival, and HNSCC cell invasion and metastasis through targeting IFIT2,^30, 32^ our results indicated that IFIT2 was unlikely to be targeted by miR-645 in CRC cells, as inhibition of miR-645 did not alter IFIT2 3′-UTR activity, nor did it impinge on the expression levels of endogenous IFIT2. Instead, miR-645 appeared to target SOX30, which has been recently reported to be a tumor suppressor in lung cancer.^23^ This was evidenced by the findings that miR-645 inhibited SOX30 3′-UTR activity and inhibition of miR-645 increased the endogenous level of SOX30 protein. In support, miR-645 expression was negatively associated with the levels of SOX30. However, overexpression of SOX30 had only a moderate, albeit statistically significant, inhibitory effect on miR-645-mediated promotion of CRC cell proliferation and sensitivity to 5-FU-induced apoptosis. This suggests that there may be mechanisms other than inhibition of SOX30 that also contribute to the oncogenic effect of miR-645 on CRC cells. It is known that the functional significance of a miR is commonly specific to tissue and cell type, and regulation of its targets is similarly highly dependent on cell type and context.^12^ In addition, miRs function as a fine-tuning mechanism in controlling gene expression.^10, 11^ While a miR potentially targets multiple mRNAs, a mRNA may in turn be regulated by a group of miRs.^34^ It is conceivable that, in addition to SOX30 mRNA, miR-645 targets mRNAs that similarly play roles in CRC cell proliferation and survival. Regardless, the results from this study have clearly shown that miR-645 is an oncogenic regulator in colon cancer.

Of note, reduction in CRC cell proliferation caused by miR-645 inhibition is associated with G1/G0 cell cycle arrest and upregulation of p21 and p27. It is known that SOX30 transcriptionally activates p53 in lung cancer,^23^ which would conceivably result in upregulation of 21, and may also have a role in upregulation of p27, leading to cell cycle arrest.^36^ However, whether this is responsible for the inhibitory effect on CRC cell proliferation when miR-645 is inhibited remains to be studied. Similarly, although our results demonstrated that sensitization of CRC cells to cisplatin and 5-FU-induced killing was associated with caspase-3 activation and was blocked by inhibition of caspase cascade, how caspase-3 is activated remains unknown. Nevertheless, results from our study showed that this was associated with the mitochondrial apoptotic pathway, as overexpression of Mcl-1 inhibited apoptosis triggered by cisplatin and 5-FU when miR-645 was inhibited.^27, 28, 29^

Genes encoding miRs are commonly found at fragile sites in the genome where alterations of genome often occur.^13, 14, 15, 16^ In colon cancer, copy number gain or loss is frequently found in particular chromosomal regions, implying that such changes may be tumourigenic. We have previously demonstrated that a segment of chromosome 17p13.1 where miR-497 mapped was frequently lost in CRC cells, which was responsible for downregulation of miR-497 that had a tumor suppressive role.^16^ In this study, we found that a fragment of chromosome 20q13.13 to which miR-645 is located frequently had copy number gain, indicating that upregulation of miR-645 is due to its copy number increases. This finding was then validated by the result that CRC samples with copy number gain of the 48–49.6 M segment showed higher expression levels of miR-645. Collectively, this suggests that copy number gain of the segment at chromosome 20q13.13 may be involved in colon cancer pathogenesis, whereas miR-645 may play a role as an oncogenic driver. Of note, chromosomal instability is commonly found during the development from colon adenoma to adenocarcinoma, including copy number gain in a number of chromosomes, including 20q.^37^ This may explain our finding that the miR-64 expression level is higher in high-grade compared with low-grade adenomas.

The functional significance of miR-645 in promoting survival and proliferation of CRC cells is confirmed by its upregulation in CRC tissues. In support, introduction of miR-645 mimics to normal colon epithelial cells enhanced proliferation and triggered anchorage-independent cell growth, consistent with the notion that the increase in miR-645 may occur at the early stage during colon cancer development. MiR-645 inhibition retarded CRC growth *in vivo*, indicating that inhibition of miR-645 may be a promising strategy in CRC treatment.

## Materials and methods

### Cell culture

Human CRC cell lines (Caco-2, Lim1215, Colo205, SW620, HCT116, SW480, Lim1863, EB and WiDr) and FHC, the human colon epithelial cell line (ATCC CRL-1831), were purchased and cultured as described previously.^16, 26, 38^ Individual cell line authentication was carried out every 6 months. The STR profile of cancer cell line were matched with the ATCC’s online databases.^39^

### Human tissue samples

Human tissue samples were from patients undergoing surgical removal of sporadic CRCs in Shanxi Cancer Hospital and Institute under the approval of Human Research Ethics Committee of the hospital. The consent form was signed by each participant. Tissue samples were processed as described previously.^16^ Extraction of proteins from crude tissue samples was carried out using the T-PER Tissue Protein Extraction Reagent as described before (Thermo Fisher Scientific, Scoresby, VIC, Australia).^40^ Clinicopathological features of patients involved are listed in Supplementary Table 3.

### miR microarray

miR microarray was performed as described previously^41, 42^ by using CapitalBio (Capital-Bio Corp., Beijing, China). The mirVANA miRNA isolation kit was used to extract total RNA (Ambion, Austin, TX, USA). Purified RNA was then labeled with Cy3 followed with hybridization on miR microarray chip (Agilent Corp., Folsom, CA, USA). miRs with expression (Mean fold change) more than 1.2-fold or lower than 0.8 fold were excluded. The data analysis was described previously.^16^

### MiR analysis using qPCR

qPCR analysis of miR expression was carried out as described before.^16^ Following specific primers were used to perform qPCR: RT-miR-645: 5′-GTCGTATCCAGTGCAGGGTCCGAGGTATTCGCACTGGATACGACTCAGCAGTA-3′ RT-U6: 5′-AACGCTTCACGAATTTGCGT-3′ qPCR-miR-645 forward, 5′-GTGCAGGGTCCGAGGT-3′ reverse, 5′-CTAGGCTGGTACTGCTGA-3′ qPCR-U6 forward, 5′-AACGCTTCACGAATTTGCGT-3′ U6 reverse, 5′-CTCGCTTCGGCAGCACA-3′. The housekeeping U6 was included as a control.

### Copy number variations (CNV) analysis

Copy number variation analysis by qPCR analysis was described before.^16^ The primers used to test CNV of the 48–49.6 M segment of chromosome 20q13.13 were: forward, 5′-TGAAATGAGTCGGCAGGTCG-3′ reverse, 5′-GCTGTGGCAAAGGGGATGTA-3′ the housekeeping gene HBB (included as a control) forward, 5′-ACACAACTGTGTTCACTAGC-3′ reverse, 5′-CAACTTCATCCACGTTCACC-3′.

### Array comparative genomic hybridization (aCGH)

aCGH array was performed as described before.^16^ NimbleScan and SignalMap softwares were used to analyze the aCGH data (Roche Diagnostics, Mannheim, Germany). Normalized and log2-transformed ratios were used to define gain or loss scoring thresholds.

### Plasmid vectors and transfection

The pCMV6-AC-GFP-Mcl-1, pCMV6-AC-GFP-SOX30 and pCMV6-AC-GFP constructs as well as the empty vector were transfected with Lipofectamine 2000 (Invitrogen, Mulgrave, VIC, Australia). These constructs contain only the coding sequences without 3′-UTRs and are therefore not subjected to regulation by miRs targeting 3′-UTRs.

### Colon cancer xenograft model

The male athymic nude BALB/c mice (5–6-week-old) were from Model Animal Research Centre of Nanjing University, China under the approval from Animal Research Ethics Committees of the hospital. CRC cells transduced with or without miR-645 inhibition were xenografted subcutaneously. Tumor growth was monitored and mice were sacrificed at 28 days after transplantation. No randomization or blinding was involved.

### Statistical analysis

Power and Sample Size calculation (PS Program) and G*Power software were employed to calculate the sample size for *in vitro* and *in vivo* studies, respectively. The data presented are as mean±s.e. of three individual experiments. The statistical significance between two selected groups were analyzed by the two-tailed Student’s *t*-test with the assumption of normal distribution of the data and equal sample variance. The statistical significance among multiple groups was examined by ANOVA. Pearson’s correlation analysis was applied to examine the association between relative abundance of the segment of Chromosome 20q13.13 and miR-645 in human CRC cell lines. All materials and results of this study were included for statistical analysis. No randomization, blinding nor exclusion of the data points was used.
