# Supplementary material for: MicroRNA-645 is an oncogenic regulator in colon cancer
Source: Oncogenesis. 2017 May 15;6(5):e335–. doi: 10.1038/oncsis.2017.37 (PMC5523070; doi:10.1038/oncsis.2017.37)
Supplement: Supplementary Information [file oncsis201737x1.pdf]

Supplementary Information

Supplementary Figures

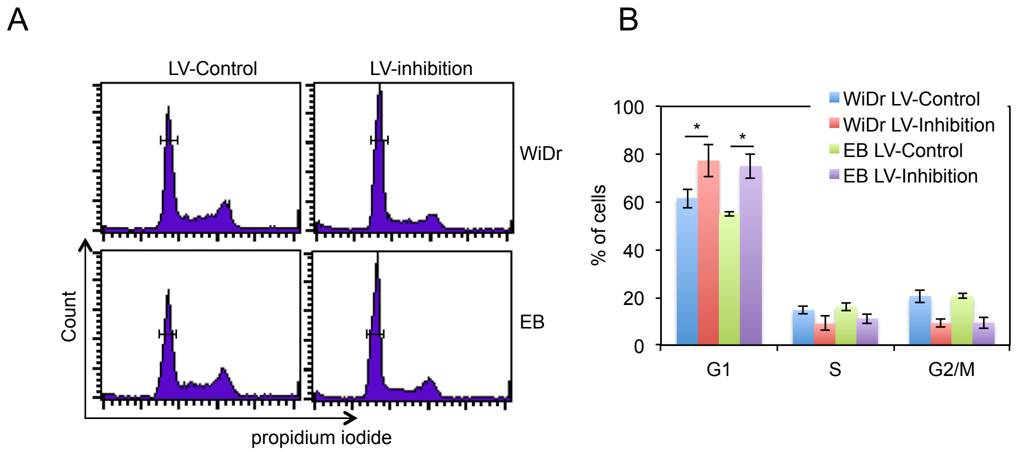

Supplementary Fig. 1. A, WiDr and EB cells were transduced with the control (LV-Control) or miR-645 inhibitors (LV-inhibition). Forty-eight hours later, cells were subjected to cell cycle analysis by propidium iodide staining using flow cytometry. The data shown are representative of three individual experiments. B, Quantitation of cell cycle distributions of WiDr and EB cells transduced with control (LV-Control) or miR-645 inhibitors (LV-inhibition). The data shown are mean ± s.e. of three individual experiments. *P<0.05, Student's t-test.

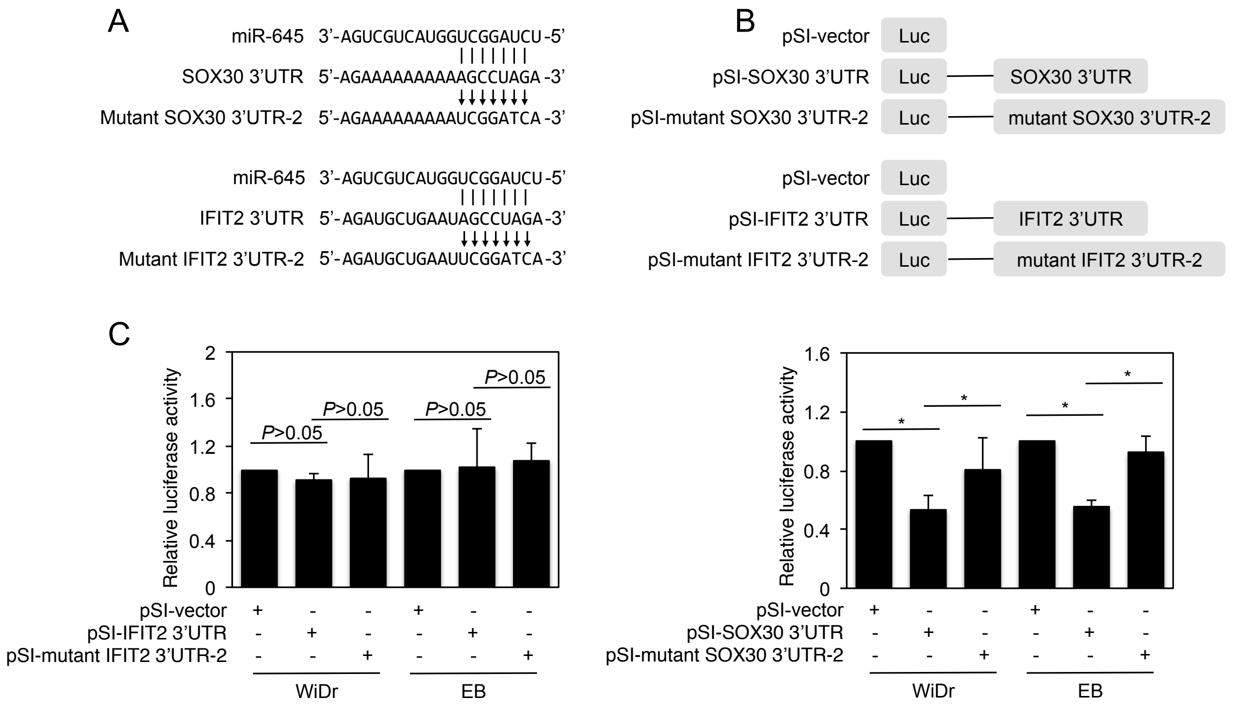

Supplementary Fig. 2. A, A schematic illustration of base-paring between miR-645 and the 3'UTR of SOX30 (Upper panel) and IFIT2 (Lower panel). Substitution of seven consecutive bases (AGCCUAG to UCGGATC) at the 3'UTR of SOX30 or IFIT2 for mutant reporter constructs is also shown. B, A schematic illustration of the pSI-CHECK2-based luciferase reporter constructs used for examining the effect of miR-645 on the 3'UTR of SOX30 or IFIT2. C, WiDr and EB cells were co-transfected with the indicated reporter constructs and renilla luciferase plasmids. Twenty-four hours later, the reporter activity was measured using luciferase assays. The data shown are mean ± s.e. of three individual experiments. *P<0.05, Student's t-test.

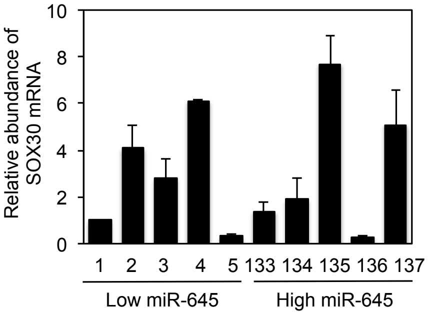

Supplementary Fig. 3. Total RNA extracts from crude colon cancer tissues that expressed relatively low (#1-5) or high (#133-137) levels of miR-645 as shown in Figure 1C were subjected to qPCR analysis of SOX30expression. The abundance of SOX30 in sample #1 was arbitrarily designated as 1. The data shown are mean ± s.e. of three individual qPCR analyses.

Supplementary Tables

Supplementary Table 1. Clinicopathological features of 5 patients whose samples were used for the miR array analysis

	Gender	Age	Anatomic location of primary tumors	TNM stage	Regional lymph node metastasis	Distant metastasis
Case 1	male	69	Rectum	II	N0	M0
Case 2	male	58	Sigmoid colon	II	N0	M0
Case 3	female	62	Sigmoid colon	II	N0	M0
Case 4	male	38	Ascending colon	III	N2	M0
Case 5	female	65	Sigmoid colon	II	N0	M0

Supplementary Table 2. Summary of miRs that were significantly altered in expression in colon cancer tissues compared with paired adjacent normal mucosa

		Folds of changes							
	miRs	Case 1	Case 2	Case 3	Case 4	Case 5	Average change	Relevance to CRC in literature	Supplementary References
Upregulated miRs in CRC tissues relative to corresponding normal mucosa	hsa-miR-135b	10.01	14.99	2.72	36.01	8.70	14.49	Promoting CRC progression by acting as a downstream effector of oncogenic pathways	1,2,3
	hsa-miR-96	5.63	1.53	9.39	16.68	37.67	14.18	Promoting CRC cell proliferation	3,4
	hsa-miR-224	5.27	16.99	2.22	6.03	11.62	8.42	A biomarkers for screening and early diagnosis of CRC	5
	hsa-miR-424	9.49	9.84	6.04	2.99	7.84	7.24	Promoting CRC progression by targeting Rictor	4, 6, 7
	hsa-miR-183	4.62	1.77	6.78	7.95	21.98	8.62	Promoting CRC cell proliferation	4, 8, 9
	hsa-miR-645	3.12	3.20	3.64	3.31	3.68	3.39		10
	hsa-miR-592	10.36	1.72	8.06	1.92	18.20	8.05	Promoting CRC progression and metastasis by targeting FoxO3A	4,11
	hsa-miR-18a	5.14	2.68	4.24	4.71	10.05	5.36	Inhibiting liver metastasis of CRC	4,12
	hsa-miR-7	1.80	5.37	5.40	2.54	15.50	6.12	Inhibiting CRC proliferation and inducing apoptosis by targeting YY1	4,13
Downregulated miRs in CRC tissues relative to corresponding normal mucosa	hsa-miR-1	2.02	4.38	67.99	257.61	18.32	70.06	Inhibiting CRC cells survival by targeting NAIP	14, 15
	hsa-miR-133b	2.19	4.80	58.54	97.67	18.32	36.30	Inhibiting CRC cells proliferation by targeting TBPL1	16,17
	hsa-miR-145	1.74	2.76	21.99	41.98	9.22	15.54	Inhibiting CRC cells survival by targeting NAIP, sensitizing CRC cells to cetuximab	18, 19, 20
	hsa-miR-143	1.38	2.04	20.42	48.74	8.70	16.26	Sensitizing CRC cells to cetuximab	20
	hsa-miR-133a	1.92	3.44	28.84	14.00	8.11	11.26	Inhibiting CRC cells proliferation, sensitizing CRC cells to doxorubicin and oxaliplatin by targeting RFFL	21
	hsa-miR-718	1.17	2.94	24.74	4.71	35.18	13.75		
	hsa-miR-363	1.56	1.12	23.20	20.83	13.75	12.09	Inhibiting CRC cells proliferation by targeting REG4	22
	hsa-miR-630	1.22	2.58	24.07	3.65	19.65	10.24		
	hsa-miR-1225-5p	3.40	2.27	9.67	3.56	12.37	6.25		
	hsa-miR-195	1.40	3.13	8.57	19.47	3.03	7.12	Sensitizing CRC cells to doxorubicin By targeting BCL2L2	7, 23
	hsa-miR-490-3p	1.51	1.32	13.13	7.13	6.18	5.85		
	hsa-miR-497	1.24	2.65	6.54	12.92	2.55	5.18	Inhibiting CRC cells proliferation and survival by targeting IGF1-R	7

Supplementary Table 3. Summary of expression of miR-645 in CRC samples with different clinicopathological groups

		Cases	Upregulation of miR-645	P value
Gender	Male	82	62	0.385#
	Female	55	39	
Age at diagnosis	≤59*	63	45	0.943#
	≥59	74	56	
Anatomic location	Ascending colon	25	20	≥0.686#
	Transverse colon	5	4	
	Descending colon	6	3	
	Sigmoid colon	24	20	
	Rectum	77	58	
TNM stage	I-II	58	40	0.093#
	III-IV	79	61	
Regional lymph node metastasis	No	60	31	≥0.062#
	N1	40	35	
	N2/N3	37	35	
Distant metastasis	M0	123	92	0.881#
	M1	14	9	

*patients were grouped according to the median age at diagnosis.
#The two-tailed Student's *t*-test was used to analyze the differences for statistical significance between two selected groups with the assumption of normal distribution of data and equal sample variance. The differences for statistical significance between multiple groups were examined by ANOVA.

Supplementary Materials and Methods

Antibodies and reagents

Antibodies against Mcl-1 (sc-12756), SOX30 (sc-20104), IFIT2 (sc-82641), and GAPDH (sc-47724) were purchased from Santa Cruz Biotechnology (Santa Cru, CA, USA). Antibodies against p27 (554069) and PARP (551025) were purchased from BD Biosciences (Bioclone, Marrickville, NSW, Australia). The antibody against p21 (05-345) was from Millipore (Billerica, MA). The antibody against Caspase-3 (ADI-APP-113) was from Enzo Life Sciences (Dural, NSW, Australia). The cell-permeable general caspase inhibitor Z-Val-Ala-Asp(OMe)-CH2F (z-VAD-fmk) (CAS 187389-52-2) was purchased from Calbiochem (Merck KGaA, Darmstadt, Germany).

BrdU proliferation assays

BrdU cell proliferation assays were carried out using the BrdU Cell Proliferation Assay kit (Cell Signaling, Arundel, QLD, Australia) as described before.[24] Briefly, cells were seeded at $5x10^3$ cells per well in 96-well plates overnight before treatment as desired. BrdU (10 μM) was added and cells were incubated for 4 hours before BrdU assays were carried out. Absorbance was read at 450 nm using a Synergy™ 2 multi-detection microplate reader (BioTek, VT).

Clonogenic assays

Clonogenic assays were carried out as described previously.[25] In brief, cells were seeded at 2000 cells/well onto 6-well culture plates. Cells were then allowed to grow for a further 12 days before fixation with methanol and staining with crystal violet (0.5% solution).

Three dimensional (3D) culture

3D culture was performed using the hanging drop technique as previously described.[26] Briefly, 500 cells were seeded into the Perfecta3D® hanging drop plate (3D Biomatrix, Ann

Arbor, MI), and the 3D spheroids were monitored with the Axiovert and Axioplan Zeiss microscope (Carl Zeiss, North Ryde, NSW, Australia) for at least 12 days. Cells were then stained with calcein AM and ethidium homodimer-1 (live/death cell viability kit; Life Technologies, Scoresby, VIC, Australia) for 24 hours. Spheroids were harvested onto slides, and images taken with a fluorescence microscope (Carl Zeiss).

Anchorage-independent cell growth

$5x10^4$ FHC cells transduced with control or miR-645 mimics were seeded in 0.3% cell agar layer, which was on top of 0.6% base agar layer in 12-well culture plates. Cells were then incubated for a further 30 days at 37^oC and 5% CO_2. Cell colony formation was then examined under a light microscope.

Apoptosis

Staining with Annexin V and Propidium iodide was carried out as described elsewhere.[27] In brief, cells were collected, washed twice with cold PBS and re-suspended in Annexin V binding buffer. Cells were then incubated with FITC-conjugated Annexin V and PE-conjugated Propidium iodide for 15 min in the dark followed by addition of binding buffer. Cells were analysed by flow cytometry within 1 hour.

Western blot analysis

Western blot analysis was carried out as described previously.[28] The intensity of bands was quantitated relative to corresponding GAPDH bands with the Bio-Rad VersaDoc™ image system (Bio-Rad, Regents Park, NSW, Australia).

Luciferase reporter assays

SOX30-3'UTR, IFIT2-3'UTR, SOX30-3'UTR-mut, or IFIT2-3'UTR-mut were constructed into pSI-CHECK2-report plasmid (Promega). Plasmids were transfected into cells (2.5×10^5) using DharmaFECT Duo Transfection Reagent (Thermo Fisher Scientific). The luciferase

activity was measured using the Dual Luciferase Reporter Assay System (Promega) by Synergy™ 2 multi-detection microplate reader (BioTek, VT). Fold-activation values were measured relative to the levels of Renilla luciferase activity in cells transfected with negative control oligonucleotides and normalized by luciferase activities.

MiR stable overexpression and inhibition

shMIMIC Lentiviral microRNAs for human miR-645 (shMIMIC-hsa-miR-645) or the corresponding control (shMIMIC-Control) were provided by Millennium Science Pty Ltd. (Mulgrave, VIC, Australia). Lentiviral vectors for inhibitor of human miR-645 (LV-hsa-miR-645 inhibition) or the control vector (LV-Control) were provided by Genechem (Shanghai, China). Cells were transduced with shMIMIC-hsa-miR-645 or LV-hsa-miR-645 inhibition according to the manufacturer's protocol.

Supplementary References

1. Valeri N, Braconi C, Gasparini P, Murgia C, Lampis A, Paulus-Hock V, *et al*. MicroRNA-135b promotes cancer progression by acting as a downstream effector of oncogenic pathways in colon cancer. *Cancer Cell*. 2014;25:469-483.

2. Khatri R, Subramanian S. MicroRNA-135b and Its Circuitry Networks as Potential Therapeutic Targets in Colon Cancer. *Front Oncol.* 2013;3:268.

3. Gaedcke J, Grade M, Camps J, Søkilde R, Kaczkowski B, Schetter AJ, *et al*. The rectal cancer microRNAome--microRNA expression in rectal cancer and matched normal mucosa. *Clin Cancer Res.* 2012;18:4919-4930.

4. Zhang Q, Ren W, Huang B, Yi L, Zhu H. MicroRNA-183/182/96 cooperatively regulates the proliferation of colon cancer cells. *Mol Med Rep.* 2015;12:668-674.

5. Zhu Y, Xu A, Li J, Fu J, Wang G, Yang Y, *et al*. Fecal miR-29a and miR-224 as the noninvasive biomarkers for colorectal cancer. *Cancer Biomark.* 2016;16:259-264.

6. Oneyama C, Kito Y, Asai R, Ikeda J, Yoshida T, Okuzaki D, *et al*. MiR-424/503-mediated Rictor upregulation promotes tumor progression. *PLoS One.* 2013;8:e80300.

7. Guo ST, Jiang CC, Wang GP, Li YP, Wang CY, Guo XY, *et al*. MicroRNA-497 Targets Insulin-Like Growth Factor 1 Receptor and Has a Tumour Suppressive Role in Human Colorectal Cancer. *Oncogene.* 2013;32:1910–1920.

8. Bi DP, Yin CH, Zhang XY, Yang NN, Xu JY. MiR-183 functions as an oncogene by targeting ABCA1 in colon cancer. *Oncol Rep.* 2016;35:2873-2879.

9. Huangfu L, Liang H, Wang G, Su X, Li L, Du Z, *et al*. miR-183 regulates autophagy and apoptosis in colorectal cancer through targeting of UVRAG. *Oncotarget.* 2016;7:4735-4745.

10. Mullany LE, Herrick JS, Wolff RK, Buas MF, Slattery ML. Impact of polymorphisms in

microRNA biogenesis genes on colon cancer risk and microRNA expression levels: a population-based, case-control study. *BMC Med Genomics.* 2016;9:21.

11. Fu Q, Du Y, Yang C, Zhang D, Zhang N, Liu X, *et al*. An oncogenic role of miR-592 in tumorigenesis of human colorectal cancer by targeting Forkhead Box O3A (FoxO3A). *Expert Opin Ther Targets*. 2016;20:771-782.

12. Teng Y, Mu J, Hu X, Samykutty A, Zhuang X, Deng Z, *et al*. Grapefruit-derived nanovectors deliver miR-18a for treatment of liver metastasis of colon cancer by induction of M1 macrophages. *Oncotarget.* 2016;7:25683-25697.

13. Zhang N, Li X, Wu CW, Dong Y, Cai M, Mok MT, *et al*. microRNA-7 is a novel inhibitor of YY1 contributing to colorectal tumorigenesis. *Oncogene.* 2013;32:5078-5088.

14. Xu X, Wu X, Jiang Q, Sun Y, Liu H, Chen R, *et al*. Downregulation of microRNA-1 and microRNA-145 contributes synergistically to the development of colon cancer. *Int J Mol Med.* 2015;36:1630-1638.

15. Migliore C, Martin V, Leoni VP, Restivo A, Atzori L, Petrelli A, *et al*. MiR-1 downregulation cooperates with MACC1 in promoting MET overexpression in human colon cancer. *Clin Cancer Res.* 2012;18:737-747.

16. Hu G, Chen D, Li X, Yang K, Wang H, Wu W. miR-133b regulates the MET proto-oncogene and inhibits the growth of colorectal cancer cells in vitro and in vivo. *Cancer Biol Ther.* 2010;10:190-197.

17. Xiang KM, Li XR. MiR-133b acts as a tumor suppressor and negatively regulates TBPL1 in colorectal cancer cells. *Asian Pac J Cancer Prev.* 2014;15:3767-3772.

18. Wang W, Ji G, Xiao X, Chen X, Qin WW, Yang F, *et al*. Epigenetically regulated miR-145 suppresses colon cancer invasion and metastasis by targeting LASP1. Oncotarget. 2016;7:68674-68687.

19. Qin J, Wang F, Jiang H, Xu J, Jiang Y, Wang Z. MicroRNA-145 suppresses cell migration and invasion by targeting paxillin in human colorectal cancer cells. *Int J Clin Exp Pathol*. 2015;8:1328-1340.

20. Gomes SE, Simões AE, Pereira DM, Castro RE, Rodrigues CM, Borralho PM. miR-143 or miR-145 overexpression increases cetuximab-mediated antibody-dependent cellular cytotoxicity in human colon cancer cells. *Oncotarget.* 2016;7:9368-9387.

21. Dong Y, Zhao J, Wu CW, Zhang L, Liu X, Kang W, *et al*. Tumor suppressor functions of miR-133a in colorectal cancer. *Mol Cancer Res.* 2013;11:1051-1060.

22. Kawasaki Y, Matsumura K, Miyamoto M, Tsuji S, Okuno M, Suda S, *et al*. REG4 is a transcriptional target of GATA6 and is essential for colorectal tumorigenesis. *Sci Rep.* 2015;5:14291.

23. Wang X, Wang J, Ma H, Zhang J, Zhou X. Downregulation of miR-195 correlates with lymph node metastasis and poor prognosis in colorectal cancer. *Med Oncol.* 2012;29:919-927.

24. Guo ST, Chi MN, Yang RH, Guo XY, Zan LK, Wang, CY, *et al*. INPP4B is an oncogenic regulator in human colon cancer. *Oncogene.* 2016;35:3049–3061.

25. Lai F, Guo ST, Jin L, Jiang CC, Wang CY, Croft A, *et al*. Cotargeting histone deacetylases and oncogenic BRAF synergistically kills human melanoma cells by necrosis independently of RIPK1 and RIPK3. *Cell Death Dis.* 2013;4:e655.

26. Wang CY, Guo ST, Wang JY, Yan XG, Farrelly M, Zhang YY, *et al*. Reactivation of ERK and Akt confers resistance of mutant BRAF colon cancer cells to the HSP90 inhibitor AUY922. *Oncotarget.* 2016;7:49597-49610.

27. Yang F, Tay KH, Dong L, Thorne RF, Jiang CC, Yang E, *et al*. Cystatin B inhibition of TRAIL-induced apoptosis is associated with the protection of FLIP(L) from degradation by the E3 ligase itch in human melanoma cells. *Cell Death Differ.* 2010;17:1354-1367.

28. Dong L, Jiang CC, Thorne RF, Croft A, Yang F, Liu H, *et al*. Ets-1 mediates upregulation of Mcl-1 downstream of XBP-1 in human melanoma cells upon ER stress. *Oncogene.* 2011; 30:3716-3726.
